# Association of serum folate with prevalence of non-alcoholic fatty liver disease among adults (NHANES 2011–2018)

**DOI:** 10.3389/fnut.2023.1141156

**Published:** 2023-04-11

**Authors:** Baodong Yao, Xiaojing Lu, Lai Xu, Yun Jiang

**Affiliations:** Department of Performance Management, Shuguang Hospital, Shanghai University of Traditional Chinese Medicine, Shanghai, China

**Keywords:** folate, non-alcoholic fatty liver disease, NHANES, dose–response, fatty liver index

## Abstract

**Background:**

Folate was involved in oxidative stress, hepatic lipid metabolism and chronic hepatic inflammation. However, evidence about the association between serum folate level and non-alcoholic fatty liver disease (NAFLD) in general population is scarce. This study aimed to explore the relationship between serum folate level and NAFLD among adults.

**Methods:**

7,146 adult participants aged 20 years and over who have complete data of serum folate level and liver function biomarkers in NHANES 2011–2018 were included. Serum folate level was measured by isotope-dilution high-performance liquid chromatography coupled to tandem mass spectrometry (LC–MS/MS). And suspected NAFLD was defined according to the United States fatty liver index (USFLI). Logistic regression and the restricted cubic spline models were performed.

**Results:**

Serum folate level was inversely associated with the presence of NAFLD. When comparing the second, third and fourth quartiles of serum folate level to the lowest quartile, the adjusted ORs of the presence of NAFLD were 0.62 (0.49–0.78), 0.65 (0.51–0.84), and 0.43 (0.32–0.56) respectively (*p* for trend<0.001). The non-linear and L-shaped relationship was found between serum folate level and the presence of NAFLD in the restricted cubic spline regression (*p* for non-linearity <0.01). Consistent with serum total folate, serum 5-Methyltetrahydrofolate level was also inversely associated with the presence of NAFLD.

**Conclusion:**

Higher serum folate level may be negatively associated with NAFLD.

## Background

Non-alcoholic fatty liver disease (NAFLD) is a major chronic liver disease with the estimated prevalence of approximately 25% in the general population worldwide (1). NAFLD is characterized by an accumulation of lipid in the hepatic parenchyma (in more than 5% of hepatocytes) independent of significant alcohol consumption, use of steatogenic drugs or hereditary diseases. NAFLD contains a range of chronic liver disorders, such as simple hepatic steatosis, nonalcoholic hepatitis (NASH), and liver fibrosis (2, 3). Although the pathogenesis of NAFLD is not fully established, oxidative stress, fat metabolism, liver inflammation, insulin resistance and some nutritional and lifestyle factors have been shown to play key roles in it (4, 5). At present, no approved effective drug for NAFLD treatment was available, and the lifestyle modification remains the most effective measures of long-term treatment for these patients with NAFLD (6).

Folate as a water-soluble B9 vitamin is a unique catalytic substrate for one-carbon transfer reactions (7, 8). Folate was involved in oxidative stress, hepatic lipid metabolism and chronic hepatic inflammation in experimental studies (8–11), which have been considered to be risk factors for NAFLD. However, the previous epidemiological studies that evaluated the association between folate and NAFLD yielded mixed findings. A small study observed that lower serum folate level was associated with severe NAFLD in 43 obese female subjects (12). Another study including 83 NASH patients found a significant correlation between low serum folate level with the histological severity of NASH (13). However, a cross-sectional study including 30 patients with NAFLD and 24 healthy controls showed similar serum folate level between NAFLD and controls groups (14). Thus, considering limited studies with small sample size, the association between serum folate level and NAFLD needs to be further explored.

Recently, one noninvasive and accurate index-the United States fatty liver index (USFLI) developed using NHANES III data was used to define NAFLD (15). The USFLI had a relatively high accuracy when used in NHANES database and have been investigated in several epidemiology studies (16, 17). Therefore, this study aimed to explore the associations of serum folate level with prevalence of NAFLD defined by USFLI among adults based on the 2011–2018 National Health and Nutrition Survey (NHANES).

## Methods

### Study subjects

NHANES is a national representative survey of the cross-sectional design which include non-institutionalized US civilians. The National Center for Health Statistics of the Centers for Disease Control (Atlanta, GA, United States) conducted the study. The National Center for Health Statistics’ Ethics Review Board approved the protocols of NHANES. Participants were selected through a stratified, multi-stage, sampling design in NHANES. All participants provide written informed consent.

**Figure 1:**
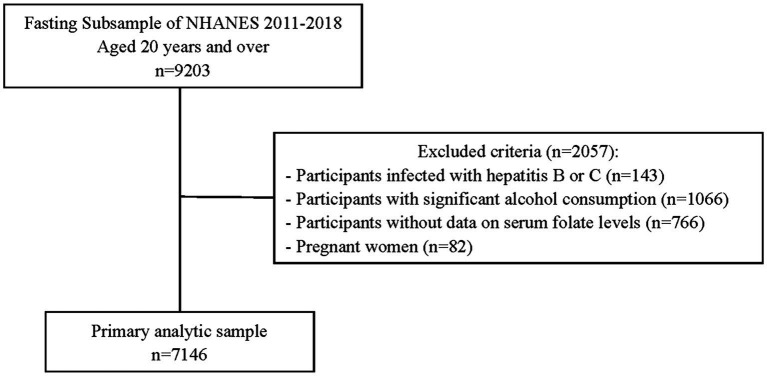
Flowchart of the sample selection.

Data from four NHANES cycles (2011–2012, 2013–2014, 2015–2016, and 2017–2018 cycles) were used. This study initially included 9,203 participants aged 20 years and over who were scheduled for a fasting blood draw and had blood tested for glucose, insulin and gamma glutamyl transferase (GGT). Participants were also excluded if (1) they are with the hepatitis B surface antigen or hepatitis C antibodies (*n* = 143), (2) they have significant alcohol consumption (>30 g/day for men and > 20 g/day for women) (*n* = 1,066), (3) women who were pregnant (*n* = 82), and (4) their data of serum folate level are missing (*n* = 766). Ultimately this study included 7,146 participants, comprising 3,310 males and 3,836 females (Figure 1).

### Definition of NAFLD

Suspected NAFLD was defined according to USFLI which was developed using NHANES data (15). The formula of USFLI was: USFLI = (e^-0.8073 × non-Hispanic black + 0.3458 × Mexican American + 0.0093 × age + 0.6151 × ln (gamma glutamyl transpeptidase) + 0.0249 × waist circumference + 1.1792 × ln (insulin) + 0.8242 × ln (glucose) -14.7812^) / (1 + e ^−0.8073 × non-Hispanic black + 0.3458 × Mexican American + 0.0093 × age + 0.6151 × ln (gamma glutamyl transpeptidase) + 0.0249 × waist circumference + 1.1792 × ln (insulin) + 0.8242 × ln (glucose) -14.7812^) × 100 (16). According to the recommended values in the previous studies (16, 17), NAFLD was defined as USFLI>30 in this study.

### Serum folate measurement

In NHANES, five folate forms (5-Methyltetrahydrofolate, pteroylglutamic acid, tetrahydrofolate, 5-formyl-tetrahydrofolate and 5,10-methenyl-tetrahydrofolate) were measured by LC–MS/MS (18). The assay is performed by combining serum specimen with an ammonium formate buffer and an internal standard mixture. Sample extraction and clean-up is performed by automated 96-probe solid phase extraction (SPE) using 96-well phenyl SPE plates and take ~1 h for a 96-well plate. Folate forms are separated within 6 min using isocratic mobile phase conditions. Quantitation is based on peak area ratios interpolated against a five-point aqueous calibration curve.

Serum total folate was calculated by adding the above five folate forms. 5-Methyltetrahydrofolate is the main bioactive form of serum total folate. Therefore, in the analyses we included serum total folate and 5-Methyltetrahydrofolate levels.

### Covariates

Based on the previously related researches (16, 17, 19), a variety of covariates were included in this study. Demographic characteristics on age (continuous), sex (men or women), race/ethnicity, education level, and family economic level were evaluated. Race/ethnicity was categorized as Mexican American, other Hispanic, non-Hispanic White, non-Hispanic Black, and other race. According to the poverty income ratio (PIR), family economic level was classified into high (PIR ≥ 4), middle (1 < PIR < 4) and low (PIR ≤ 1). Education was dichotomized as high school or below versus above high school. Behavioral and lifestyle factors included smoking status, drinking status, and recreational activity. Individuals were classified into never smoker if they have never smoked or smoked <100 cigarettes in life, current smoker if they have smoked ≥100 cigarettes in life and were currently smoking, and former smoker if they have smoked ≥100 cigarettes in life and were currently no longer smoking. Drinking status was considered as yes (Ever have 4/5 or more drinks every day) or no. Recreational activity was categorized as vigorous, moderate, or not. Body mass index (BMI) was calculated as weight divided by height squared in kg/m^2^ (under/normal weight: <25 kg/m^2^; overweight: 25–30 kg/m^2^; and obese ≥30 kg/m^2^). Diabetes was defined according to one of the following four criteria: self-reported prior diagnosis of diabetes, taking any antidiabetic medication, glycosylated hemoglobin measurement ≥6.5%, or fasting plasma glucose level ≥ 126 mg/dL. Participants with hypertension were defined as taking hypertension medication, self-reported prior diagnosis of hypertension, systolic blood pressure ≥ 130 mmHg, or diastolic blood pressure ≥ 80 mmHg (20). Total energy and fat intakes were calculated by averaging energy and fat intakes collected during two 24-h total nutrient recall interviews. To account for the potential influence of cholesterol, we also adjusted for serum total cholesterol.

### Statistical analysis

Descriptive statistics were conducted to characterize the individuals. The continuity variables were reported as the mean ± standard deviation (SD), and the categorical variables were reported as number and percentages. Differences between the group with NAFLD and the group without NAFLD were determined by chi-square tests for categorical variables and by *t*-test for continuity variables. Logistic regression was used to examine the association of serum total folate and 5-Methyltetrahydrofolate levels in quartiles with NAFLD, and covariates as mentioned above were included in the adjusted analyses. The adjusted odds ratios (ORs) were calculated and the lowest quartile of serum total folate and 5-Methyltetrahydrofolate level was taken as the reference group. To evaluate potential nonlinear associations between serum total folate and 5-Methyltetrahydrofolate and the risk of having NAFLD, restricted cubic spline analyses were conducted (21). Three knots located at the 5th, 50th and 95th percentiles of serum total folate and 5-Methyltetrahydrofolate levels were used in the restricted cubic spline functions in this study. And *p* values for non-linearity were calculated by testing the null hypothesis that the coefficient of the second spline was equal to 0. We also conducted subgroup analyses by sex, given the differences in the prevalence rate of NAFLD for male and female. In sensitivity analyses, we additionally adjusted for the levels of serum triglyceride and low-density lipoproteins cholesterol (LDL-C), which were measured in a fasting subsample of participants in NHANES.

According to analytical guidelines of NHANES, appropriate sampling weights should be used considering the stratified, multistage probability sample design. And the new weights were created for the combined NHANES (2011–2018) cycles. All statistical data were analyzed using Stata version 12.0 (Stata Corporation, College Station, TX, United States). Statistical significance was defined as two-sided *p* ≤ 0.05.

## Results

The characteristics of the study population grouped by the presence or absence of NAFLD are shown in Table 1. Compared with participants without NAFLD, those with NAFLD were older (53.39 years vs. 47.78 years), more likely to be obese (71.47% vs. 22.29%) and had a higher prevalence of diabetes (37.89% vs. 10.48%) and hypertension (70.27% vs. 46.25%). Participants with NAFLD also had a higher prevalence of low education level and low family income. The daily fat intake in NAFLD participants were significantly higher than that in participants without NAFLD.

**Table 1:** Population characteristics by the presence or absence of NAFLD determined by USFLI in NHANES 2011–2018.

Characteristics	Overall (*n* = 7,146)	NAFLD determined by USFLI		
		No (*n* = 4,657)	Yes (*n* = 2,489)	value of *p*
**N**				
Age (years)	49.73 ± 17.56	47.78 ± 17.82	53.39 ± 16.46	<0.001
Sex, *n* (%)				<0.001
Male	3310 (46.32)	2043 (43.87)	1267 (50.90)	
Female	3836 (53.68)	2614 (56.13)	1222 (49.10)	
Race/ethnicity, *n* (%)				<0.001
Mexican american	1,009 (14.12)	445 (9.56)	564 (22.66)	
Other hispanic	807 (11.29)	495 (10.63)	312 (12.54)	
Non-hispanic White	2676 (37.45)	1694 (36.38)	982 (39.45)	
Non-hispanic Black	1460 (20.43)	1141 (24.50)	319 (12.82)	
Other race	1194 (16.71)	882 (18.94)	312 (12.54)	
Levels of education, *n* (%)				<0.001
≤high school	3215 (45.01)	1954 (41.98)	1261 (50.68)	
>high school	3928 (54.99)	2701 (58.02)	1227 (49.32)	
Economic status, *n* (%)				<0.001
Low (PIR ≤ 1)	1506 (23.22)	942 (22.24)	564 (25.08)	
Middle (1 < PIR < 4)	3434 (52.95)	2215 (52.29)	1219 (54.20)	
High (PIR ≥ 4)	1545 (23.82)	1079 (25.47)	466 (20.72)	
Recreational activities, *n* (%)				<0.001
Other	3693 (51.70)	2199 (47.23)	1494 (60.07)	
Moderate	1878 (26.29)	1210 (25.99)	668 (26.86)	
Vigorous	1572 (22.01)	1247 (26.78)	325 (13.07)	
Smoking status, *n* (%)				<0.001
Never smoked	4212 (58.99)	2842 (61.11)	1370 (55.04)	
Current smoker	1268 (17.76)	867 (18.64)	401 (16.11)	
Former smoker	1660 (23.25)	942 (20.25)	718 (28.85)	
Drinking status, *n* (%)				<0.001
No	6434 (90.04)	4257 (91.41)	2177 (87.46)	
Yes	712 (9.96)	400 (8.59)	312 (12.54)	
Body mass index, *n* (%)				<0.001
Under/normal weight	2067 (28.99)	1958 (42.13)	109 (4.39)	
Overweight	2253 (31.60)	1654 (35.59)	599 (24.13)	
Obese	2810 (39.41)	1036 (22.29)	1774 (71.47)	
Diabetes, *n* (%)				<0.001
Non-diabetic	5715 (79.97)	4169 (89.52)	1546 (62.11)	
Diabetic	1431 (20.03)	488 (10.48)	943 (37.89)	
Hypertension, *n* (%)				<0.001
No	3243 (45.38)	2503 (53.75)	740 (29.73)	
Yes	3903 (54.62)	2154 (46.25)	1749 (70.27)	
**Daily dietary intake**				
Energy (kcal/d)	1973.60 ± 777.62	1968.50 ± 789.79	1983.13 ± 754.39	0.449
Total fat intake (g/d)	77.19 ± 36.88	76.48 ± 36.98	78.52 ± 36.67	<0.05
Serum total cholesterol (mg/dL)	189.07 ± 40.95	188.14 ± 40.64	190.80 ± 41.46	<0.01
Serum total folate (nmol/L)	42.76 ± 28.27	43.08 ± 24.51	42.17 ± 34.20	0.192
Serum 5-Methyltetrahydrofolate (nmol/L)	40.12 ± 26.35	40.52 ± 22.93	39.39 ± 31.77	0.086

NAFLD, non-alcoholic fatty liver disease; USFLI, US fatty liver index.

Data were summarized as mean ± SD for continuous variables or as a numerical proportion for categorical variables.

**Figure 2:**
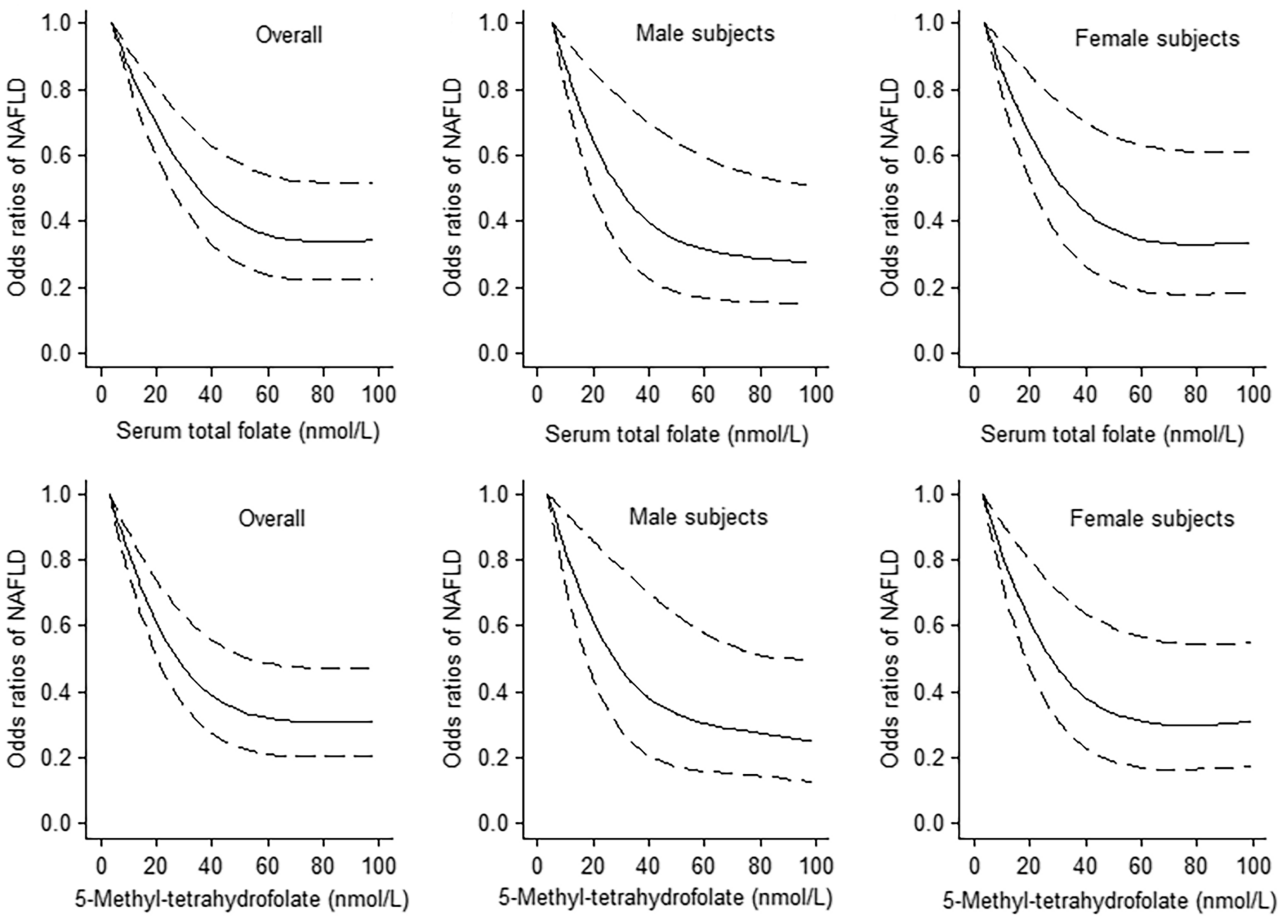
The dose–response analyses between serum total folate and 5-Methyltetrahydrofolate levels with non-alcoholic fatty liver disease (NAFLD) using the restricted cubic spline. The middle line and upper and lower line represent the estimated odds ratios and its 95% confidence interval, respectively. The lowest values of serum total folate and 5-Methyltetrahydrofolate levels were used as reference.

The results of logistic regression analyze between serum total folate and 5-Methyltetrahydrofolate with NAFLD are exhibited in Table 2. The serum total folate levels were negatively associated with the presence of NAFLD. When comparing the second, third and fourth quartiles of serum folate level to the lowest quartile, the adjusted ORs of the presence of NAFLD were 0.62 (0.49–0.78), 0.65 (0.51–0.84), and 0.43 (0.32–0.56), respectively (*p* for trend<0.001) (Table 2). The non-linear and L-shaped relationship was found between serum total folate level with the presence of NAFLD in the restricted cubic spline regression (*p* for non-linearity <0.01) (Figure 2). Similar results were found in the subgroup analysis stratified by sex (Table 3 and Figure 2). Consistent with serum total folate, serum 5-Methyltetrahydrofolate levels were also inversely associated with the presence of NAFLD (Table 2 and Figure 2). The results were generally consistent in sensitivity analyses additionally adjusting for the levels of serum triglyceride and LDL-C in a fasting subsample of participants (Supplementary Table S1).

**Table 2:** Associations between serum total folate and 5-Methyltetrahydrofolate levels with NAFLD in NHANES 2011–2018.

Item	NAFLD determined by USFLI	
	OR (95% CI)	value of *p*
**Serum total folate (nmol/L)**		
Quartile 1 (≤ 25.30)	Ref.	
Quartile 2 (25.30–36.60)	0.62 (0.49–0.78)	<0.001
Quartile 3 (36.60–54.03)	0.65 (0.51–0.84)	<0.01
Quartile 4 (>54.03)	0.43 (0.32–0.56)	<0.001
*p* for trend		<0.001
**Serum 5-Methyltetrahydrofolate (nmol/L)**		
Quartile 1 (≤ 23.38)	Ref.	
Quartile 2 (23.38–34.50)	0.62 (0.48–0.79)	<0.001
Quartile 3 (34.50–51.50)	0.65 (0.50–0.84)	<0.01
Quartile 4 (>51.50)	0.41 (0.31–0.55)	<0.001
*p* for trend		<0.001

NAFLD, non-alcoholic fatty liver disease; USFLI, US fatty liver index.

The following covariates were adjusted: age, sex, race/ethnicity, education level, family economic level, smoking status, drinking status, recreational activity, body mass index, diabetes, hypertension, serum total cholesterol, and total dietary intakes of energy and fat.

**Table 3:** Associations between serum total folate and 5-Methyltetrahydrofolate levels with NAFLD stratified by gender in NHANES 2011–2018.

Item	NAFLD determined by USFLI	
	OR (95% CI)	value of *p*
**Male**		
*Serum total folate (nmol/L)*		
Quartile 1 (≤ 24.38)	Ref.	
Quartile 2 (24.38–34.50)	0.63 (0.42–0.94)	0.025
Quartile 3 (34.50–49.40)	0.58 (0.40–0.85)	<0.01
Quartile 4 (>49.40)	0.37 (0.24–0.57)	<0.001
*p* for trend		<0.001
*Serum 5-Methyltetrahydrofolate (nmol/L)*		
Quartile 1 (≤ 22.48)	Ref.	
Quartile 2 (22.48–32.60)	0.62 (0.41–0.95)	0.028
Quartile 3 (32.60–47.10)	0.55 (0.39–0.80)	<0.01
Quartile 4 (>47.10)	0.36 (0.23–0.56)	<0.001
*p* for trend		<0.001
**Female**		
*Serum total folate (nmol/L)*		
Quartile 1 (≤ 26.10)	Ref.	
Quartile 2 (26.10–38.50)	0.60 (0.43–0.85)	0.004
Quartile 3 (38.50–57.70)	0.68 (0.47–0.98)	0.039
Quartile 4 (> 57.70)	0.38 (0.25–0.58)	<0.001
*p* for trend		<0.001
*Serum 5-Methyltetrahydrofolate (nmol/L)*		
Quartile 1 (≤ 24.10)	Ref.	
Quartile 2 (24.10–36.40)	0.58 (0.41–0.82)	0.003
Quartile 3 (36.40–55.10)	0.60 (0.41–0.86)	0.007
Quartile 4 (> 55.10)	0.37 (0.25–0.56)	<0.001
*p* for trend		<0.001

NAFLD, non-alcoholic fatty liver disease; USFLI, US fatty liver index.

The following covariates were adjusted: age, race/ethnicity, education level, family economic level, smoking status, drinking status, recreational activity, body mass index, diabetes, hypertension, serum total cholesterol, and total dietary intakes of energy and fat.

## Discussion

In this large study of 7,146 adult participants from NHANES 2011–2018, serum total folate and 5-Methyltetrahydrofolate levels were inversely associated with the presence of NAFLD determined by USFLI in adults. Meanwhile, the dose–response relationship between serum total folate and 5-Methyltetrahydrofolate levels and NAFLD was non-linear.

To the best of our knowledge, this is the first observational study to investigate the association between serum folate level and NAFLD defined by USFLI in a general population. The results of previous research with relatively small sample sizes or specific population were inconsistent. A one-center, cross-sectional study including 30 biopsy-proven NAFLD patients and 24 healthy controls showed that serum folate levels were similar in two groups (14). However, in a study including 43 obese (BMI ≥ 35 kg/m^2^) participants, lower serum folate level was found in patients with NAFLD compared to those participants with normal liver or minimal alterations (12). In addition, a hospital-based study including 83 patients with biopsy-proven NASH observed that low serum folate level was significantly associated with the histological severity of NASH (13). A study of Chinese adults with NAFLD (70 subjects determined by a liver biopsy and 130 subjects determined by H-MRS) also found that serum folic acid level was negatively correlated with liver steatosis grades (22). A recent study using data from NHANES 2017–2018 found that higher serum folate level was associated with lower prevalence of metabolic dysfunction-associated fatty liver disease (MAFLD) (19). In our study, we investigated the associations between serum folate level and NAFLD in general population. Our results about the significant negative association between serum total folate level and the presence of NAFLD in the general population were consistent with previous studies. Moreover, a non-linear relationship with L-shape were apparent between serum folate level with NAFLD, and the level of serum total folate up to 60 nmol/L was the threshold at which the dose–response line started to plateau. These results suggest important public health and clinical implications for the management and prevention of NAFLD patients.

The underlying mechanisms of inverse association between serum folate level and NAFLD may be explained through different aspects. Folate, as well as folic acid has powerful antioxidant functions in human health, which was capable of directly scavenging reactive oxygen species (ROS) (23). Oxidative stress was implicated in the pathogenesis of NAFLD (24), likely through promoting inflammation (25). In addition, several clinical studies have shown lower serum folate levels were associated an increase in BMI and involved in insulin resistance (12, 26), which has been considered to be the risk factors of NAFLD. Third, a few experimental studies demonstrated that folic acid supplementation could prevent NAFLD by activating the AMPK signaling pathways in the rodent model (27). AMPK as a key regulator of metabolism plays an important role in regulating hepatic lipogenesis (28).

The primary strength of this study was utilization of a large sample size from a nationally representative sample of NHANES. In NHANES there were detailed data on lifestyle, diet, and medical comorbidities, and we adjusted for a large number of covariates that made the results of this study more stable and credible. Moreover, the non-linear dose–response relationship between serum folate level and NAFLD was clearly elucidated using the restricted cubic splines in this study. However, there are a few of limitations that need to be considered. First, reverse causation may be the concern due to the cross-sectional nature of NHANES. Second, in this study noninvasive index (USFLI) but not a liver biopsy was used to identify NAFLD, which might result in the misclassification of NAFLD. However, The USFLI was developed using NHANES data, and the previous studies have demonstrated that the USFLI had a high accuracy (29).

## Conclusion

Our results suggest that serum folate level was inversely associated with the prevalence of NAFLD in the general population. Since this study is the first attempt to investigate the association between serum folate level and NAFLD defined by USFLI, more future longitudinal and intervention studies are needed.

## Data availability statement

The raw data supporting the conclusions of this article will be made available by the authors, without undue reservation.

## Ethics statement

NHANES was approved by the National Center for Health Statistics Research Ethics Review Board.

## Author contributions

BY performed the statistical analyses, and wrote the manuscript. XL and LX contributed to interpretation of the data and the manuscript draft. YJ conceived the study and reviewed the manuscript. All authors contributed to the article and approved the submitted version.

## Funding

This work was supported by the National Natural Science Foundation of China (no. 81403237), Three-year Action Plan of Shanghai to Accelerate the Development of Traditional Chinese Medicine (no. ZY (2018–2020)-RCPY-2005), and Research project of Shanghai University of Traditional Chinese Medicine for COVID-19 infection (no. 2022YJ-18).

## Conflict of interest

The authors declare that the research was conducted in the absence of any commercial or financial relationships that could be construed as a potential conflict of interest.

## Publisher’s note

All claims expressed in this article are solely those of the authors and do not necessarily represent those of their affiliated organizations, or those of the publisher, the editors and the reviewers. Any product that may be evaluated in this article, or claim that may be made by its manufacturer, is not guaranteed or endorsed by the publisher.
