# Supplementary material for: Association of serum folate with prevalence of non-alcoholic fatty liver disease among adults (NHANES 2011–2018)
Source: Front Nutr. 2023 Apr 11;10:1141156. doi: 10.3389/fnut.2023.1141156 (PMC10126427; doi:10.3389/fnut.2023.1141156)
Supplement: Supplementary file 1 [file Table_1.PDF]

Supplementary Table 1 Associations between serum total folate and 5-Methyltetrahydrofolate levels with NAFLD in a fasting subsample of participants in NHANES 2011-2018

Item	NAFLD determined by USFLI	
	OR (95% CI)	*p-value*
**Serum total folate (nmol/L)**		
Quartile 1 (≤ 26.20)	Ref.	
Quartile 2 (26.20-37.90)	0.71 (0.47-1.08)	0.108
Quartile 3 (37.90-55.90)	0.64 (0.46-0.91)	<0.05
Quartile 4 (>55.90)	0.38 (0.25-0.58)	<0.001
P for trend		<0.001
**Serum 5-Methyltetrahydrofolate (nmol/L)**		
Quartile 1 (≤ 24.00)	Ref.	
Quartile 2 (24.00-35.45)	0.67 (0.46-0.97)	<0.05
Quartile 3 (35.45-53.20)	0.66 (0.47-0.93)	<0.05
Quartile 4 (>53.20)	0.37 (0.25-0.55)	<0.001
P for trend		<0.001

Abbreviation: NAFLD, non-alcoholic fatty liver disease; USFLI, US fatty liver index.

The following covariates were adjusted: age, sex, race/ethnicity, education level, family economic level, smoking status, drinking status, recreational activity, body mass index, diabetes, hypertension, serum total cholesterol, serum triglyceride, serum low-density lipoproteins cholesterol, and total dietary intakes of energy and fat.
